# Natural Plant-Derived Compounds in Food and Cosmetics: A Paradigm of Shikonin and Its Derivatives

**DOI:** 10.3390/ma16124377

**Published:** 2023-06-14

**Authors:** Sonia Malik, Patrycja Brudzyńska, Muhammad Rehan Khan, Oksana Sytar, Abdullah Makhzoum, Alina Sionkowska

**Affiliations:** 1 Laboratory of Woody Plants and Crops Biology (LBLGC), University of Orleans, 45067 Orléans, France; 2 Department of Biomaterials and Cosmetic Chemistry, Faculty of Chemistry, Nicolaus Copernicus University in Toruń, Gagarin 7, 87-100 Toruń, Poland; 3 Department of Agricultural Science, University of Naples Federico II, Via Università 133, 80055 Portici, Italy; 4 Institute of Plant and Environmental Sciences, Faculty of Agrobiology and Food Resources, Slovak University of Agriculture, 949 76 Nitra, Slovakia; 5 Department of Biological Sciences & Biotechnology, Botswana International University of Sciences and Technology, Palapye 10071, Botswana

**Keywords:** antioxidant properties, bioactive compounds, *Boraginaceae*, food additives, food packaging, herbal products, natural colorants, nutraceuticals, pigments, plant natural products

## Abstract

Shikonin and its derivatives are the natural naphthoquinone compounds produced in the roots of the Boraginaceae family. These red pigments have been used for a long time in coloring silk, as food colorants, and in the Chinese traditional system of medicines The resurgence of public interest in natural and plant-based products has led to this category of compounds being in high demand due to their wide range of biological activities including antioxidant, antitumor, antifungal, anti-inflammatory ones. Different researchers worldwide have reported various applications of shikonin derivatives in the area of pharmacology. Nevertheless, the use of these compounds in the food and cosmetics fields needs to be explored more in order to make them available for commercial utilization in various food industries as a packaging material and to enhance their shelf life without any side effects. Similarly, the antioxidant properties and skin whitening effects of these bioactive molecules may be used successfully in various cosmetic formulations. The present review delves into the updated knowledge on the various properties of shikonin derivatives in relation to food and cosmetics. The pharmacological effects of these bioactive compounds are also highlighted. Based on various studies, it can be concluded that these natural bioactive molecules have potential to be used in different sectors, including functional food, food additives, skin, health care, and to cure various diseases. Further research is required for the sustainable production of these compounds with minimum disturbances to the environment and in order to make them available in the market at an economic price. Simultaneous studies utilizing recent techniques in computational biology, bioinformatics, molecular docking, and artificial intelligence in laboratory and clinical trials would further help in making these potential candidates promising alternative natural bioactive therapeutics with multiple uses.

## 1. Introduction

Shikonin is a natural dye that has been used for several centuries for coloring clothes, as a food pigment, and as traditional Chinese medicine. It is a bioactive compound with potential applications in the food, cosmetics, textile, and pharmaceutical industries [1,2]. Shikonin and its derivatives are the red pigments extracted from the roots of the Boraginaceae family, which includes different genera such as *Arnebia*, *Alkanna*, *Anchusa*, *Echium*, *Lithospermum,* and *Onosma*. These are commercially important naphthoquinones that were identified for the first time in *L. erythrorhizon Siebold & Zucc.* (commonly known as zicao). The molecular formula of shikonin is C_16_H_16_O_5_. Shikonin derivatives are specialized lipophilic compounds derived from precursors formed from the shikimate and mevalonate pathways [3]. They are well known for their broad spectrum of biological activities including anticancer, antioxidant, antiviral, and anti-inflammatory ones and recently as anti-COVID-19 agents [4,5,6]. They have been used as a natural source for coloring silk, as an ingredient in cosmetics, and as a food additive. The public interest in plant-based natural products due to cultural practices and natural adaptability with the human body has led to this compound being in high demand. For instance, despite the high price of a biolipstick (USD 30 a stick) made up of shikonin, about 2 million sticks were sold within a few days by Kanebo, a Japanese cosmetics company in 1985. The biolipstick comprising the natural colorant shikonin was not only biologically prepared but also possessed antimicrobial properties against bacteria and other micro-organisms [7]. The production of these pigments in roots requires the plants to grow for three to four years in their natural habitat. Due to the resurgence of consumer interest in natural bioactive compounds, the demand for these potent compounds has been continuously rising, and to fulfill the growing need, different biological systems have been employed by adopting various biotechnological methods [2,8,9]. It is worth mentioning that shikonin was the first commercial product produced by Mitsui Petrochemical Industries Ltd. (now Mitsui Chemicals Inc.), Tokyo, Japan, from *Lithospermum erythrorhizon* using in vitro culture technology. Different reviews have appeared in this regard during the last decade [2,10]. All these articles have focused on various emerging issues such as the establishment of reliable production systems to meet market demand, functional identification, and the future clinical development of shikonin and its derivatives against various diseases. For instance, the implications of shikonin as a natural bioactive molecule to treat various cancers and its anticancer mechanisms have been reviewed by Wang et al. [11]. Andujar and his co-workers presented the synthesis and the pharmacological properties of shikonin [1]. The various natural sources, distribution, biosynthesis, isolation, pharmacology, and stability or toxicity factors have been explained by Yadav et al. [10]. Malik et al. described the different biotechnological techniques to improve the production of these compounds under in vitro conditions [2]. Nevertheless, their potential in food and cosmetic applications has not been reviewed so far. Therefore, the aim of the present article is to highlight the recent advances in the field of shikonin-derivative research and their applications in the area of food technology, cosmetics, and pharmacological industries. The properties of shikonin, which make them interesting candidates for various food-related uses such as food storage and packaging, for curing diseases, and as cosmetics for the skin, have been discussed in detail. This review was carried out based on reports published between 2002 and 2023 by employing scientific search engines such as PubMed, Scopus, and Science Direct. The inclusion criteria were members of Boraginaceae family, shikonin derivatives, and biological properties.

## 2. The Properties of Shikonin

### Antimicrobial Activities against Food-Borne Pathogens

**Figure 1:**
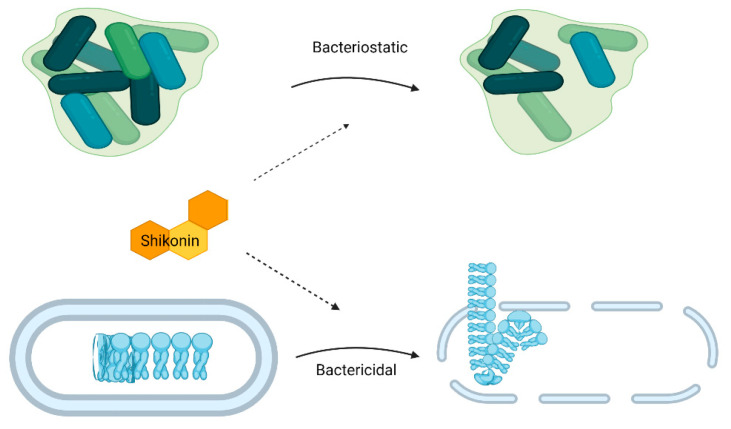
Antibacterial action of shikonin and its derivatives against food-borne pathogens (created with BioRender.com).

An ideal food additive is one that should be able to exert an antimicrobial effect against food-borne pathogens (i.e., bacteria and fungi). Shikonin is a major bioactive naphthoquinone extracted from the roots of *Lithospermum erythrorhizon* and has been reported to possess excellent antimicrobial activity against common food-borne pathogens i.e., *Staphylococcus aureus*, *Listeria monocytogenes, Enterococcus faceium*, *Bacillus subtilis*, and *Candida albicans* [12,13]. Generally, shikonin and its derivatives display better antibacterial activity against Gram-positive bacteria and various species of lactic acid bacteria than against Gram-negative bacteria (since shikonin is hydrophobic in nature and thus has more affinity for Gram-positive bacteria due to their lack of an outer membrane) [1]. The antibacterial property of shikonin might be due to both bactericidal and bacteriostatic effects (Figure 1). This bactericidal phenomenon was confirmed by Lee et al. [14]; the authors reported complete disintegration of the cytoplasmic membrane, the release of cytoplasmic contents, and cell lysis. On the other hand, shikonin inhibited biofilm formation by *L. monocytogenes* by regulating quorum sensing, autoregulatory alternative sigma factor SigB, and flagellar formation by exerting its bacteriostatic mechanism [12]. The minimum inhibitory concentrations (MICs) of shikonin and its derivatives were reported to be between 1.5 and 200 µg/mL against different bacterial strains [13,15]. For instance, Wan et al. [15] reported that the MIC of shikonin against different strains of *S. aureus* ranged from 35 µg/mL to 70 µg/mL. Furthermore, shikonin inhibited bacterial growth by hyperpolarizing the cell membrane, disrupting cell membrane integrity, reducing intracellular ATP concentration, and changing the cellular morphology. On the other hand, a difference in MICs for the same *S. aureus* strain (ATCC 25923) from two different studies was observed [14,15], which could be due to the difference in protocols used; however, it is worth mentioning that further studies are required to determine the correct MICs of shikonin against bacterial strains.

There is less consensus regarding the antifungal ability of shikonin and its derivatives in the literature. For instance, the MIC of shikonin and its derivatives is observed to be between 0.1 and 100 µg/mL against different fungal species, including *Candida albicans*, *Aspergillus* sp., *Penicillium chrysogenum*, and *Rhizoctonia phaseoli* [16,17,18]. Generally, shikonin can exert its antifungal activity by modulating the expression of certain genes, i.e., the upregulation of adenylyl-cyclase-associated protein, which can cause the rearrangement of cytoskeleton organization, and the generation of endogenous reactive oxygen species (ROS) due to elevation of cAMP, which can decrease mitochondrial membrane potential; the upregulation of DPP3, which is a key gene for farnesol (antibiofilm molecule in *Candida* spp.) biosynthesis; the downregulation of hypha and adhesion specific genes, i.e., the HWP1 and ALS family; the downregulation of metabolism-related genes, including fermentation- (ALD5 and ADH1) and glycolysis-related genes (CDC19 and HXK2); the downregulation of antioxidant-defense-related genes (SOD2 and SOD5); and by the accumulation of endogenous nitric oxide or by a reduction in the deacetylation process of H3K56 by reduced levels of NAM [16,17,19,20]. For instance, a recent study reported the modulation of 27 metabolites related to nitrogen metabolism, oxidative stress, lipid synthesis, amino acid synthesis, glycolysis, histone deacetylation, and the tricarboxylic acid cycle [17]. Furthermore, shikonin has also demonstrated the ability to be effective against drug-resistant strains of *C. albicans* [19]. The antimicrobial influence of shikonin and its derivatives along with their MICs is presented in Table 1.

**Table 1:** Antimicrobial influence of shikonin against food-borne pathogens.

Type of Microbe	MIC (µg/mL)	Antimicrobial Mechanism	References
**Bacteria**			
*L. monocytogenes* strains (ATCC 19115, ATCC 15313, A17, A24, B9, B19, C6, and C34)	25–100	Several virulence-associated genes (agrA, flaA, and sigB) were influenced by shikonin	[12]
*S. aureus* strains (ATCC 29213, ATCC 25923, A86, A48, 13 S2, S4, 124, and 265)	35–70	Reduction in intracellular ATP concentration, cell membrane hyperpolarization, and destruction of cell membrane	[15]
**Fungi**			
*C. albicans* strains (SC5134, 8376, 6355, 4390, 6375, 5473, 6885, 2336, 9664, and 4647)	2–4	↑ of DPP3 gene caused an increase in farnesol production, ↓ of HWP1 gene caused detachment from the abiotic substrate	[21]
*C. albicans* strains (SC5134, SN250, cta4Δ/Δ, and yhb1Δ/Δ	4–8	↓ of YHB1 gene led to the intercellular accumulation of nitric oxide, which manifested nitrosative stress	[16]
*A. terreus*	2	↑ of adenylyl-cyclase-associated protein, which linked actin reorganization to Ras signaling led to elevated cAMP and caused ROS accumulation	[20]
*C. albicans* strains (YFC 497, YFC 803), *C. glabrata* YFC 501, *C. krusei* YFC 827, *C. tropicalis* YFC 052, *C. parapsilosis* YFC 826, *S. cerevisiae* YFC 250, *T. cutaneum* YFC 517, and *A. fumigatus* YFC 526	8–64	Shikonin and its derivative displayed better antifungal activity than fluconazole	[22]
*C. albicans* strains (Y0109, 21, 100, 103, 271, 876, 18, 38, and 953)	2–8	↓ of glycolysis-related (CDC19 and HXK2) and fermentation-related genes (ADH1 and ALD5) providing more electrons for the mitochondrial respiratory chain, which caused the accumulation of ROS	[19]
*C. albicans* SC5314	4	↑ of CaMCA1 led to mitochondrial-mediated intrinsic cell apoptosis	[23]
*C. albicans* strains (C5314, CASS1, and hst3Δ/pTET-HST3)	6–8	↓ of NAM resulted in suppression of deacetylation of H3K56	[17]

Here, ↑ = upregulation and ↓ = downregulation of a certain gene, which ultimately influences a particular pathway.

## 3. Role of Shikonin in Smart Packaging

### 3.1. Antioxidant Activity

Sufficient antioxidant activity is necessary for a food additive to be considered for inclusion in complex food systems. Oxidation reactions in food systems can cause color change, off flavors, and loss of nutritional quality; however, the presence of antioxidants can prevent that from happening or at least delay these phenomena. The antioxidant activity of shikonin is mainly due to the presence of the phenoxy group to neutralize free radicals by receiving or donating electrons [24]. It has been suggested that the -OH group at the C-1′ position positively influenced the radical scavenging properties of shikonin and its derivatives by facilitating the donation of the hydrogen atom from this carbon atom [25]. The antioxidant capacity of active films in terms of DPPH and ABTS radical scavenging activities ranged between 60 and 88%; however, some studies reported a higher antioxidant capacity for shikonin in the ABTS assay, while some reported a higher DPPH radical scavenging activity as compared to ABTS scavenging activity, which can be correlated with the nature of the bioactive and the polymer and the release of the active compound (shikonin) in water (ABTS) and alcohol (DPPH) solutions [26,27]. For instance, a higher ABTS radical scavenging activity (35–95%) for carboxymethyl cellulose (CMC)/agar-based films was observed as compared to DPPH radical scavenging activity (17–40%) due to the difference in the release of active compounds in water- and alcohol-based mediums [27].

### 3.2. Color Response Efficiency

**Figure 2:**
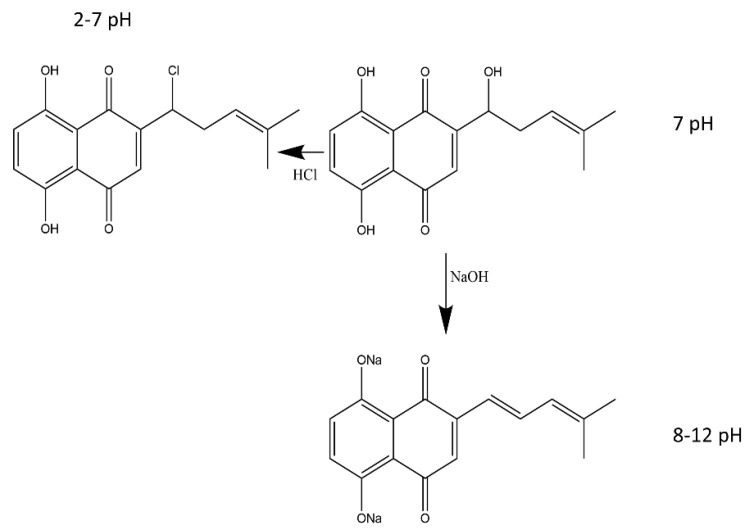
Reaction scheme of shikonin at acid–base conditions (created with Chemdraw^®^).

**Figure 3:**
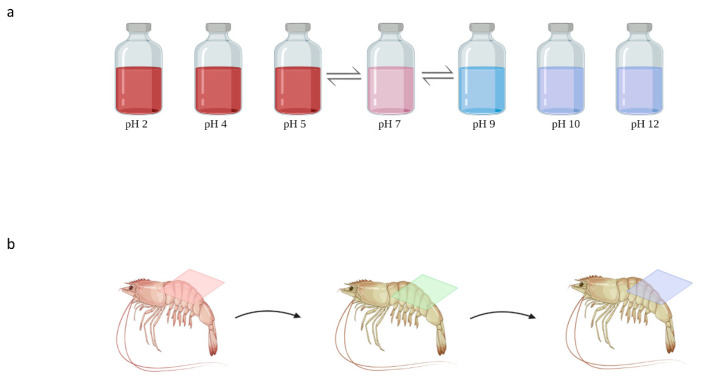
Color of shikonin solution at various pH ranges (**a**) and color change of smart packaging as an indicator of spoilage for meat products (**b**) from acidic to alkaline conditions (created with BioRender.com).

Color indicator films can exhibit different color changes corresponding to pH changes. Generally, shikonin-based active films can change color from red to blue when the pH value is increased from 2 to 12. More specifically, the color turned bright red at pH 2–7; when the pH increased from 8 to 10, the color changed from red–purple to purple and displayed blue at pH 11–12 [24,28]. Shikonin is stable under acidic conditions; however, its molecular structure quickly changes under alkaline conditions. This could be due to the dissociation of the phenolic hydroxyl group of naphthoquinone compounds due to the resonance effect caused by the alkali; additionally, the intensity of the blue color was believed to increase due to secondary dissociation of the phenolic hydroxyl group [24]. The naphthoquinone moiety of shikonin is highly unstable at alkaline pH, which decomposes quickly at a pH above 7 leading to hydrolysis and significant color change (Figure 2) [29]. The pH-sensitive color-changing function of shikonin at pH ≥ 7 is highly desirable for its use as a smart color indicator sensor. Ezati and Rhim [30] exposed cellulose paper containing shikonin to ammonia and acetic acid vapors to determine its ability as a gas sensor. The color indicator paper turned red when exposed to acetic acid and blue immediately when exposed to ammonia vapors. The volatile ammonia reacts with H_2_O present in the indicator to produce hydroxyl ions and ammonium; the hydroxyl ions then interact with the phenolic hydroxyl groups to produce a dark-blue color. Volatile compounds, i.e., trimethylamine and ammonia, are generated during the decomposition of meat and meat products; thus, shikonin-based pH-sensitive smart films can be used as sensors to detect pH changes in meat products [31]. It has been demonstrated that shikonin-based films can be effectively used as an indicator of spoilage for protein-based foods, i.e., shrimp and pork, due to the generation of TVBN (total volatile basic nitrogen) during spoilage. Furthermore, these films appear bright red when the in-contact meat product is fresh, purple when it is slightly spoiled, and bluish violet when it is completely spoiled. For instance, it has been reported that fresh shrimps had a TVBN of <12 mg/100 g (and the film displayed a magenta color); however, after 3 days of storage at 20 °C, the TVBN sharply rose to > 25 mg/100 g (and the film displayed a violet–blue color indicating inedibility and decomposition) (Figure 3) [28,32,33].

Generally, shikonin solution (around 2–7 pH) exhibits absorbance peaks at ~486, 518, and 558 nm under UV–Vis spectroscopy, and the relative intensity of the peaks reduced to 488 nm after the pH of the solution changed from 8 to 10; however, the absorbance of the peaks shifted to 586 and 624 nm at pH 11 and 12, respectively, due to the nonstable and highly sensitive nature of the chromophore molecule under alkaline conditions [28,32]. Similarly, the a*-value of the films loaded with shikonin increased slightly with an increase in pH to 7; however, a sharp decrease in the a*-value was observed, which indicates that the yellowness of the samples decreased significantly with increasing pH. The L*-value was similarly influenced by the increase in pH, with the lowest value observed at pH 10. Consequently, the ΔE value (total color difference) of the films increased linearly with increasing pH values [24]. Similarly, Roy et al. observed an increase in ΔE values from 42 to 58 with increasing pH (from 2 to 12) due to the low stability of shikonin at alkaline pH [27].

### 3.3. Release Kinetics of Shikonin

Understanding the phenomena of release kinetics of a food additive from the film into complex food systems is important since the release kinetic parameters, i.e., diffusion coefficient and partition coefficient, have a role in controlling the nutritional quality of the food products. Furthermore, it is also necessary to have an optimum concentration of an additive in the packaging material, which can exhibit the desired antioxidant/antimicrobial activity by keeping in view the regulatory constraints stipulated by food authorities [34]. The release of shikonin from the film into complex food systems was evaluated by using different food simulants (both alcohol and water based). Generally, the release rate of an additive is influenced by many factors, i.e., the solubility of the additive into the simulant, the polymer matrix’s nature and integrity, and the swelling rate of the polymer [35]. Shikonin is a hydrophobic compound and is alcohol soluble; thus, a higher release of shikonin can be expected with an increasing concentration of alcohol (from 0 to 95%) [24]. For instance, food simulants with high alcohol content (> 50%) displayed 4–6 times more release of shikonin from cellulose-based films than low alcohol content (0–10%) food simulants since the release rate of shikonin is less dependent on the swelling ratio of the matrix and more dependent on the diffusion, the solubility of shikonin, and the type of simulant [24,26,30].

## 4. Shikonin in Medicine and Pharmacy

Shikonin and its derivatives, isolated from traditional medicinal plant species of the genera *Lithospermum*, *Alkanna*, *Arnebia*, *Anchusa*, *Onosma*, and *Echium* belonging to the *Boraginaceae* family, may show various pharmacological activities and can be used in medicine and pharmacy areas [10]. Shikonin (R-enantiomer) exists as an enantiomeric pair with alkannin (S-enantiomer), hence known as A/S, and shows various pharmacological activities. Both S and A revealed relatively similar pharmacological capacities, such as antimicrobial, anticancer, antioxidant, wound healing, anti-inflammatory, and antithrombotic properties [36]. At the same time, after modification to the naphthazarin ring or side chain of shikonin, some shikonin derivatives showed better anticancer activity and lower toxicity than shikonin itself [6]. Shikonin derivatives have the potential for the treatment of medullary thyroid carcinomas as well [37]. In the literature, one can find that 18 novel shikonin derivatives have been synthesized in order to obtain compounds that exhibit a higher cytotoxicity on melanoma cells [38]. 

Shikonin has a significant role in the production of reactive oxygen species (ROS), in facilitating exosome delivery, and in initiating apoptosis [39]. Examples of these effects include modulating the PI3K/AKT/mTOR and MAPKs signaling; inhibiting the activation of TrxR1, RIP1/3, PKM2, Src, and FAK; and regulating the expression of ERP57, C-MYC, ATF2miR-128, MMPs, and GRP78 (Bip). Shikonin is able directly to target mitochondria. The endogenous ROS increase and mitochondrial aerobic respiration shift participate in the action of shikonin against the pathogenic yeast *Candida albicans* [19]. Shikonin is recommended as a supportive antifungal agent in the clinical management of *Candida albicans* biofilms [21].

At the same time, shikonin is able to cause mitochondrial dysfunction in cancer cells. Shikonin accumulation is connected with a shikonin-dependent deregulation of cellular Ca^2+^ and the level of ROS. This deregulation leads to a disintegration of the mitochondrial membrane potential, cell-cycle arrest, microtubule dysfunction, and eventually the induction of apoptosis [40]. The addition of shikonin to paclitaxel endorsed cancer cell mitotic arrest and resulted in significantly greater levels of cell apoptosis [41]. The downregulation of PFKFB2 expression is connected with shikonin’s presence, which caused inhibition of the Warburg effect and exerted antitumor activity in lung cancer cells [42]. As a potential natural inhibitor targeting PAK1 kinase, shikonin has encouraging potential in the treatment of pancreatic cancer [43].

The following pharmacokinetic parameters are typical for shikonin: It has an adverse oral bioavailability, has 64.6% of the binding rate of plasma protein, and increases level of some metabolic enzymes, especially cytochrome P450. Such parameters have been estimated by a few clinical trials [13]. Nowadays, with the development of novel drug delivery systems through the use of liposomes, nanoparticles, cyclodextrin complexes, microemulsions, nanogels, etc., the pharmaceutical innovation of shikonin is in progress with the improvement of shikonin-based drugs. It was observed that methoxy poly (ethylene glycol)-b-poly (ϵ-caprolactone) micelles significantly improved the endothelial-to-mesenchymal transition–inhibiting effect of free shikonin, which makes it suitable for development as a lipophilic drug carrier [44].

Shikonin with its anti-inflammatory potential is able to participate in the improvement of early-stage diabetic retinopathy [45], which is a major microvascular complication of diabetes. In a study with a diabetic rat model, it was shown that shikonin increases glucose uptake in skeletal muscle cells via an insulin-independent pathway dependent on calcium. In vivo and in vitro studies on the advantageous effects of shikonin on glucose metabolism found that the compound has properties that can be used for developing a novel treatment for type 2 diabetes [46].

Diabetic difficulties may appear from enhanced flux through the polyol pathway. The enzyme aldose reductase in the polyol pathway catalyzes glucose to galactitol conversion. For that reason, the inhibition of aldose reductase has been known as a crucial strategy in the prohibition and attenuation of long-term diabetic problems. A new platform in developing potent aldose reductase inhibitors such as shikonin for future therapeutic intervention against diabetic vascular complications was studied [47].

Similarly, shikonin has been reported to possess anti-inflammatory and anti-angiogenic roles and is indicated as a novel potential therapy strategy for choroidal neovascularization (CNV). CNV is a feature related to neovascular age-related macular degeneration leading to vision loss. Shikonin has been shown to alleviate CNV by acting as a pyruvate kinase M2 inhibitor in macrophages [48].

It was found that shikonin can be used as a new therapeutic agent for the treatment of allergic dermatitis including atopic dermatitis [49]; even earlier, the possible toxicological effects of shikonin, which can cause skin allergy, were discussed [13]. Mouse models of wounds confirmed that shikonin promoted wound healing of skin wounds in a mouse model [49]. The effect of shikonin on wound healing is supported through promoting the activity of cell growth and the suppression of skin inflammation via inhibitory activity on proteasomes [50]. The therapeutic effects of shikonin on skin diseases, such as psoriasis, melanoma, and hypertrophic scars, have been discussed in the scientific literature. A functional and mechanistic investigation of shikonin in scarring found that shikonin stimulates apoptosis in scar fibroblasts by differentially regulating the expression of caspase 3, Bcl-2, phospho-Erk1/2, and phospho-p38. Shikonin is able downregulate the expression of collagen I, collagen III, and alpha-smooth muscle actin genes, hence attenuating collagen synthesis in scar-derived fibroblasts [51].

## 5. Shikonin for Cosmetic Applications

### 5.1. Extraction of Shikonin and Its Derivatives for Cosmetic Applications

Shikonin is the main component of a red pigment found in the roots of various plants. The chiral pair shikonin and alkannin are nowadays used as a valuable ingredient in cosmetics and also as a colorant in Japan, while in Europe and North America, mainly alkannin is used for cosmetic and food coloration [52]. As shikonin and alkannin are found in the *Boraginaceae* family, 17 selected plants from this family were studied for their secondary metabolite content using the HPCE technique. In the extraction process, methanol was used, while for shikonin derivatives, hexane was applied. Among the tested species such as *Anchusa azurea, Anchusa undulata*, *Borago officinalis*, *Buglossoides purpureocaerulea*, *Cerinthe minor*, *Cynoglossum creticum*, *Echium italicum*, *Echium russicum*, *Echium vulgare*, *Lindelofia macrostyla*, *Lithospermum officinale*, *Nonea lutea*, *Pulmonaria mollis*, *Pulmonaria obscura*, *Omphalodes verna*, *Symphytum cordatum*, and *Symphytum officinale,* shikonin and its derivatives were only extracted from the roots of three species: *Echium italicum*, *E. russicum*, *E. vulgare* and also from *Lithospermum officinale*. Later on, these compounds were isolated from other species from the Boraginaceae family, for instance, *Arnebia euchroma*, *A. guttata*, *Onosma paniculatum*, *O. hookeri*, and obviously from *Lithospermum erythrorhizon* [53]. Shikonin and its derivatives are commonly extracted from the roots of *L. erythrorhizon*, the root tissues of which are hard and thus cause the extraction process to be more complicated. One of the possibilities to increase the extraction efficiency is an extension of the processing time conducted at high temperatures; however, this entails high energy consumption; thus, high-pressure and ultrasonic extractions gain more interest. The studies by Kim et al. concerned the determination of optimal conditions of the extraction process conducted with these two techniques in order to obtain *L. erythrorhizon* extracts valuable for cosmetic application [54]. The research provides information about the cosmetic activity of the tested extracts in relation to shikonin content and extraction yield. It was found that extraction with the application of high pressure and ultrasonication under optimized conditions of pressure, temperature, time, and ultrasonic wave intensity can improve the extract’s cosmetic activity such as skin whitening and strengthening of skin immunity. The optimal extraction conditions were as follows: 500 MPa for 60 min and 120 kHz for 90 min, leading to the highest extraction yield and shikonin content, which were equal 27.49% and 3.19% (*w*/*w*), respectively. In comparison, conventional extraction with ethanol led to an extraction yield equal to 16.32% and a 1.81% content of shikonin. The improved extraction process caused greater skin immune activation and hyaluronidase inhibition. Furthermore, the tyrosinase inhibitory activity and skin whitening activity were 20% higher for the extract obtained using extraction enhanced with ultrasonication and high-pressure techniques than for conventional extraction with ethanol [54]. In research conducted by P. Papageorgiou, olive oil was assessed as the best solvent for hydroxynaphthoquinone and isohexenylnaphthazarin (which include alkannin and shikonin) extraction from *Alkanna tinctoria* roots. Its main advantage besides nontoxicity was the fact that, at room temperature, the extract contains mainly monomeric isohexenylnaphthazarins and low-molecular-weight polymeric hydroxynaphthoquinones. As the rising temperature is the factor that causes polymerization, research indicated that extraction conducted at 40 °C led to the minimalization of the polymeric fraction, whereas a temperature equal to 60 °C caused an increase in the polymerization process. Optimization of the extraction process conditions in terms of polymerization of hydroxynaphtoquinones can affect the final biological activity of the products obtained from *Alkanna tinctoria* roots extracted with olive oil. Polymerization leads to lower solubility, deterioration of biological activity, and a red coloration shine, which limits the application of alkannin, shikonin, and their derivatives in the cosmetic, food, and pharmaceutical industry [55]. HPLC⁃PAD was assessed as a useful, precise, and repeatable method for the simultaneous determination of shikonin and its derivatives such as acetylshikonin, isobutyryl shikonin, 2⁃methylbutyryl shikonin, and many more from *Lithospermum* oil extracted from *L. erythrorhizon* by supercritical fluid extraction. The highest content of active compounds was obtained when the extraction parameters pressure, temperature, and CO_2_ flow rate were as follows: 23 MPa, 40 °C, and 27 L/h. This method ensures the stability of the active compound content and is more beneficial than the traditional one, which favors its wide usage [56]. Supercritical carbon dioxide extraction, with optimal extraction conditions equaling 400 bar and 60 °C, was also used to obtain a stable red colorant from *Lithospermum erythrorhizon*, which contains shikonin and its derivatives. Furthermore, research has shown that concentrations of up to 10% of colorant do not cause skin irritation, which was confirmed by patch tests. Color measurements by the CIE LAB chromaticity test indicated that there were no differences in results between the extracted colorant and a commercial synthetic substitute. Studies of color stability and DPPH radical scavenging activity indicated that the colorant obtained through supercritical carbon dioxide extraction was characterized by higher stability than the sample extracted with ethanol. The stability of this colorant may be promising from a cosmetic point of view [57]. One of the most important parameters of cosmetic ingredients is their stability during the production process and storage. Size-exclusion chromatography was applied to observe the influence of alkaline media and isohexenylnaphthazarin ester hydrolysis on isohexenylnaphthazarin polymerization. The study showed that polymeric alkannin/shikonin and isohexenylnaphthazarin are formed during isohexenylnaphthazarin ester hydrolysis. Nonpolar solvents are preferred for the extraction; they lead to the minimization of the formation of the polymeric isohexenylnaphthazarin, alkannin, and shikonin [58].

### 5.2. Antioxidant Activity and UV Radiation Protection Properties of Shikonin and Its Derivatives in Terms of Cosmetic Application

Shikonin and alkannin are characterized by the antioxidant activities and can protect the skin from oxidative stress for both monomeric and oligomeric compounds. Furthermore, extracts of *Alkanna tinctoria* roots were also tested, and the relationship between structure and activity was assessed. Research has shown that shikonin and alkannin and their esters presented very high radical scavenging activity, which was linked with the naphthoquinone moiety present in their structures. However, the esterification of hydroxyl groups on the side chain did not influence the measured parameter. According to the research, promising antiradical activity also was indicated for the *Alkanna tinctoria* root extract, which was obtained with organic solvents and olive oil addition at room temperature. These results suggest the great potential of the tested compounds for their use in the cosmetics industry [59]. The antioxidant activity of alkannin and shikonin and their derivatives was also examined in another study; to perform antioxidant activity measurements, oils such as olive oil, corn oil, sunflower oil, and lard were used. Shikonin showed very promising antioxidant activity when applied to lard; however, processes such as polymerization negatively affect this property. Shikonin, both monomeric and polymeric, beneficially influences olive oil quality, retarding its oxidation. Furthermore, monomeric shikonin with citric acid addition applied to sunflower oil showed moderate antioxidant properties. Research showed that alkannin, shikonin, and their derivatives when applied to oils can improve their antioxidant properties [60]. High antityrosinase activity and antioxidant activity of shikonin were also confirmed by molecular docking results and among others, ABTS, hydroxyl, superoxide, and DPPH free radical scavenging activity [61]. Research conducted by Yoo et al. suggested that *L. erythrorhizon* extract, in which derivatives of shikonin were identified, has the ability to protect skin from aging caused by oxidative stress. Authors indicated that this protection based on the inhibition of skin collagen degradation may be enhanced by the presence of phenolic compounds with antioxidant properties found in the tested extract too [62]. Another study conducted by Glynn et al. focused on skin protection based on glycation inhibition, inflammatory responses suppression, UV absorption potential, and enhancement of oxidative defenses by *L. erythrorhizon* extract and its main naphthoquinone—shikonin—in facial and lip cosmetic matrices. Research has proved that an extract containing shikonin can beneficially influence the skin [63]. Extracts of mulberry and *L. erythrorhizon* containing total anthocyanin and shikonin as colorants in a concentration equal to 4.92 mg/g and 9.58 mg/g, respectively, were examined for their protective properties against photodamage induced by UVB. Both extracts were applied to the skin of a model mouse, which was subsequently UVB irradiated. Studies have shown that a reduction in thickness of the epidermis, thickness and depth of wrinkles, erythema index, and collagen fiber damage and an increase in the water capacity were observed when the extracts were used, which confirms the potential of the applied extracts as valuable skin protection ingredients against UVB-induced photodamage [64].

### 5.3. Cosmetic Properties and Skin Sensitization Potential of Shikonin and Its Derivatives

**Figure 4:**
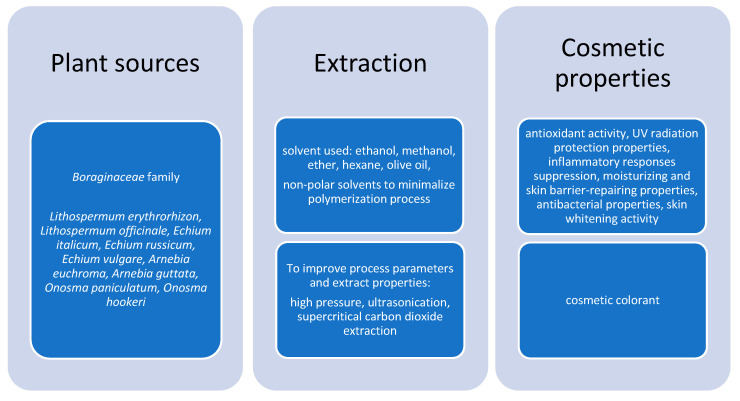
Scheme presenting plant sources, extraction solvents, and cosmetic properties of shikonin and its derivatives.

Shikonin’s properties for application in skin treatment have also been studied. The *L. erythrorhizon* root extract was investigated in terms of its moisturizing and skin-barrier-repairing properties. Bioengineering techniques were applied to measure the human skin parameters of 30 volunteers aged 20–30 years old. Extracts were obtained with ethanol, and several emulsions with different extract concentrations were prepared. Patch tests were used to observe skin irritation, and skin erythema was also tested. Studies led to the conclusion that the tested cosmetics with the extract beneficially influence skin properties due to increasing skin humidity, showing moisturizing effects and causing a decrease in transepidermal water loss, thus improving the function of the skin barrier [65]. However, during research, the selected extract was not assessed in terms of the content of shikonin and its derivatives, but another study showed that methanol extracts of *Lithospermum erythrorhizon* root contain among others shikonin, deoxyshikonin, acetylshikonin, isobutyshikonin, and acrylshikonin [66]. Takahiro and Ikuyo indicated that, in ether extracts, β-hydroxyisovalerylshikonin can be found instead of deoxyshikonin and β-acetoxyisovalerylshikonin, while the rest of the composition is very similar [67]. Authors suggested that ethanol extracts of a selected plant used to prepare cosmetic products were predicted to consist of almost the same compounds as the previously analyzed methanol or ether ones; nevertheless, authors emphasized that future studies would be essential to determine which particular ingredient is responsible for a beneficial effect on skin [65]. It is also worth mentioning that the encapsulation process with cyclodextrins can enhance the bioavailability of alkannin, shikonin, and their derivatives, which can be used in cosmetic products in the form of creams, sprays, or gels [66]. Shikonin and other natural compounds that are found in food or cosmetics were tested in terms of their skin-sensitizing potential. A local lymph node assay with an elicitation phase was used. Studies have shown that shikonin obtained positive results even at a very low concentration of 0.05% (dissolved in DMSO) causing serious reactions and was classified as an ingredient with skin-sensitizing properties [68]. Previously, a paper by Natsuaki et al. also focused on allergic responses to shikonin, which was one of the medicinal ingredients [69]. However, there are also studies that indicated shikonin’s anti-allergic activity [70,71] and normal T cell activity regulation potential [72]. Authors suggested that the wide biological activity of shikonin could affect the test results, which only emphasizes how important is to extend the bioactivity evaluation of natural compounds applied to food and cosmetics [73]. Moreover, the skin photoallergy potential of shikonin was assessed in a patch test. In the research, 5, 10, 25, and 50% concentrations were used in the patch test system for 48 h. The test was applied to 33 healthy Chinese subjects, while the photo patch test was administered to 206 subjects, whose skin was characterized by photosensitivity. It was indicated that a 5% concentration of shikonin can be applied in cosmetics products as a safe ingredient not causing photosensitive or contact allergic reactions on the skin [74]. Another property of shikonin is the ability to stimulate the growth of human keratinocytes and dermal fibroblasts, which was confirmed by MTS assay allowing for cell growth measurements; thus, shikonin can promote wound healing. A cell-based proteasome activity assay was also applied, which indicated shikonin’s inhibitory activity on the proteasome and its ability to suppress skin inflammation [50]. All the plant sources from which shikonin and its derivatives were obtained, the solvents used, and the methods applied to improve the extraction process as well as the cosmetic properties of these compounds are presented in Figure 4.

## 6. Conclusions

In light of the above literature, it is found that shikonin produced by plants and its derivatives prepared in the laboratory can be used as a natural source of bioactive molecules to be utilized in different sectors including food and cosmetic ingredients. Due to significant antimicrobial activities, these compounds could be successfully used in various cosmetic formulations and as a fresh wrap in food materials. As these compounds are produced in low quantities and under stress conditions in plants, it is important to explore other families and genera to find out better production systems. Moreover, it would be worth it to find out the alternate ways of production of shikonin derivatives with minimum disturbances to the environment. In order to achieve sustainable production at an economic price, further studies are required to be carried out using modern techniques with special emphasis on the safety and clinical aspects.

## Data Availability

There are no research data in this review paper.
